# Exercise-mediated extracellular vesicle-derived non-coding RNAs and cardiovascular protection: mechanistic insights and bibliometric analysis

**DOI:** 10.3389/fphys.2026.1945161

**Published:** 2026-09-09

**Authors:** Pengfei Yang, Xing Wang, Zhaohui Guo, Chengcheng Ji, Xinbo Zhang

**Affiliations:** 1 Department of Physical Education, Shanghai Dianji University, Shanghai, China; 2 School of Physical Education, Shanghai University of Sport, Shanghai, China; 3 School of Artificial Intelligence and Data Science, Shanghai Dianji University, Shanghai, China; 4 School of Athletic Performance, Shanghai University of Sport, Shanghai, China

**Keywords:** bibliometric analysis, cardiovascular protection, exercise, extracellular vesicle, non−coding RNAs, synergy

## Abstract

**Background:**

Exercise-mediated extracellular vesicle (EV)-derived non-coding RNAs (ncRNAs) contribute to cardiovascular protection, but a systematic synthesis of the field is lacking, and the evolution of research hotspots and knowledge structure remains unclear. This bibliometric study maps the research landscape, frontier hotspots, and emerging trends, while also interpreting the multi-target synergistic mechanisms underlying exercise-mediated cardioprotection based on published evidence.

**Methods:**

Publications were retrieved from the Web of Science Core Collection, PubMed, and Scopus from database inception to April 3, 2026. After deduplication and screening, 188 articles were included. VOSviewer, CiteSpace, and the R-based Bibliometrix package were employed for analyses of countries, institutions, journals, highly cited papers, and keywords.

**Results:**

Annual publications grew from 1 in 2013 to 28 in 2025. China (65 publications) and the United States (48 publications) together contributed over 60% of the total. Core journals included Frontiers in Physiology (11 publications), International Journal of Molecular Sciences (9 publications), and Cells (8 publications). Research evolved in three phases: early multi-directional exploration (2015–2020), a middle phase converging on EV-centric themes (2021–2023), and a recent return to fundamental biology (2023–2026). “Extracellular vesicles” ranked first in frequency (48 occurrences), while “physical activity” had the highest centrality (centrality 0.22). Ten keyword clusters were identified, with “aerobic exercise,” “animal model,” and “human cell” emerging as recent burst keywords. Mechanistically, informed by bibliometric patterns and published preclinical evidence, we propose that exercise may exert anti-apoptotic, antioxidant, anti-inflammatory, and anti-fibrotic effects by engaging a potential synergistic network involving EV-derived ncRNAs, though this framework requires experimental validation.

**Conclusion:**

Research in this field is transitioning from mechanistic exploration to clinical translation. China and the United States are the leading contributors, and the intersection of exercise intervention and EVs biology represents the most dynamic research direction. Future efforts should prioritize methodological standardization, large-scale clinical validation, and integration of artificial intelligence approaches, while addressing translational barriers in delivery, manufacturing, regulation, and clinical trial design.

## 1 Introduction

Cardiovascular diseases (CVDs) remain the leading cause of death worldwide, posing a major challenge to global public health (Martin et al., 2024). In 2023, CVD accounted for 19.2 million deaths (32% of all global deaths) and 437 million disability-adjusted life years—a 1.4-fold increase from 1990. Ischemic heart disease, ischemic stroke, intracerebral hemorrhage, and hypertensive heart disease are the predominant causes of death and disability. More than 80% of the CVD burden is attributable to modifiable risk factors, including hypertension, unhealthy diet, dyslipidemia, and physical inactivity (Global Burden of Cardiovascular Diseases and Risks 2023 Collaborators, 2025). Pharmacological and interventional therapies have improved short-term outcomes in CVD patients, but long-term survival remains suboptimal. This highlights the urgent need for novel non-invasive strategies and molecular targets for early prevention and precision therapy (Regan et al., 2025).

Extracellular vesicles (EVs) are lipid-bilayer particles secreted by multiple cell types, with small EV subtypes generally ranging from 30 to 150 nm in diameter. They carry bioactive molecules such as proteins, lipids, and non-coding RNAs (ncRNAs), and mediate intercellular communication in cardiovascular homeostasis and disease (Collado et al., 2023). MicroRNAs (miRNAs), long non-coding RNAs (lncRNAs), and circular RNAs regulate cardiomyocyte apoptosis, endothelial function, inflammatory responses, and fibrosis through post-transcriptional regulation and epigenetic modifications (Zhang et al., 2024). The lipid-bilayer structure of EVs protects their encapsulated ncRNAs from nuclease degradation, making them stable in blood and other biofluids. This stability gives them potential as both biomarkers and delivery vehicles (Eivazi et al., 2025). In cardiovascular pathophysiology, EV-derived ncRNAs have been shown to modulate angiogenesis, oxidative stress, fibrosis, and cardiomyocyte apoptosis (Zhang et al., 2023), highlighting their value for mechanistic research and therapeutic targeting in CVD.

Regular exercise is an important cornerstone of cardiovascular health, substantially reducing the risk of cardiovascular morbidity and mortality across populations (Zhang W. et al., 2026). Recent studies show that exercise promotes EV release from cardiomyocytes, endothelial cells, and skeletal muscle cells through physical stimuli like fluid shear stress and mechanical stretch. This is accompanied by significant changes in the expression profiles of miRNAs, lncRNAs, and other ncRNAs within these EVs (Kong et al., 2026; Gao and Yu, 2026). For cardiovascular protection, preclinical studies have reported that exercise may trigger the production and release of endogenous EV-derived ncRNAs. These molecules may engage multiple targets and signaling pathways to potentially boost myocardial tolerance against ischemic and hypoxic stress. These findings offer a new molecular explanation for exercise-mediated cardioprotection and highlight the exercise–EV–derived ncRNAs axis as a promising non-invasive intervention strategy (Hou et al., 2019; Akhmerov and Parimon, 2022).

Despite these advances, existing evidence comes mainly from animal studies and small-sample clinical investigations, with considerable methodological heterogeneity across reports. The knowledge structure, research hotspots, and collaboration patterns in this field have not been systematically examined through bibliometric approaches. Traditional reviews are largely qualitative and cannot quantify trends or identify emerging directions. Bibliometric analysis, which integrates publication trends, collaboration networks, and keyword co-occurrence data, can objectively map the development of a discipline. Accordingly, we performed a bibliometric analysis to map the research landscape, identify core clusters and paradigm transitions, and provide an overview that may help inform future mechanistic and translational studies (Chen et al., 2026).

## 2 Materials and methods

**Figure 1:**
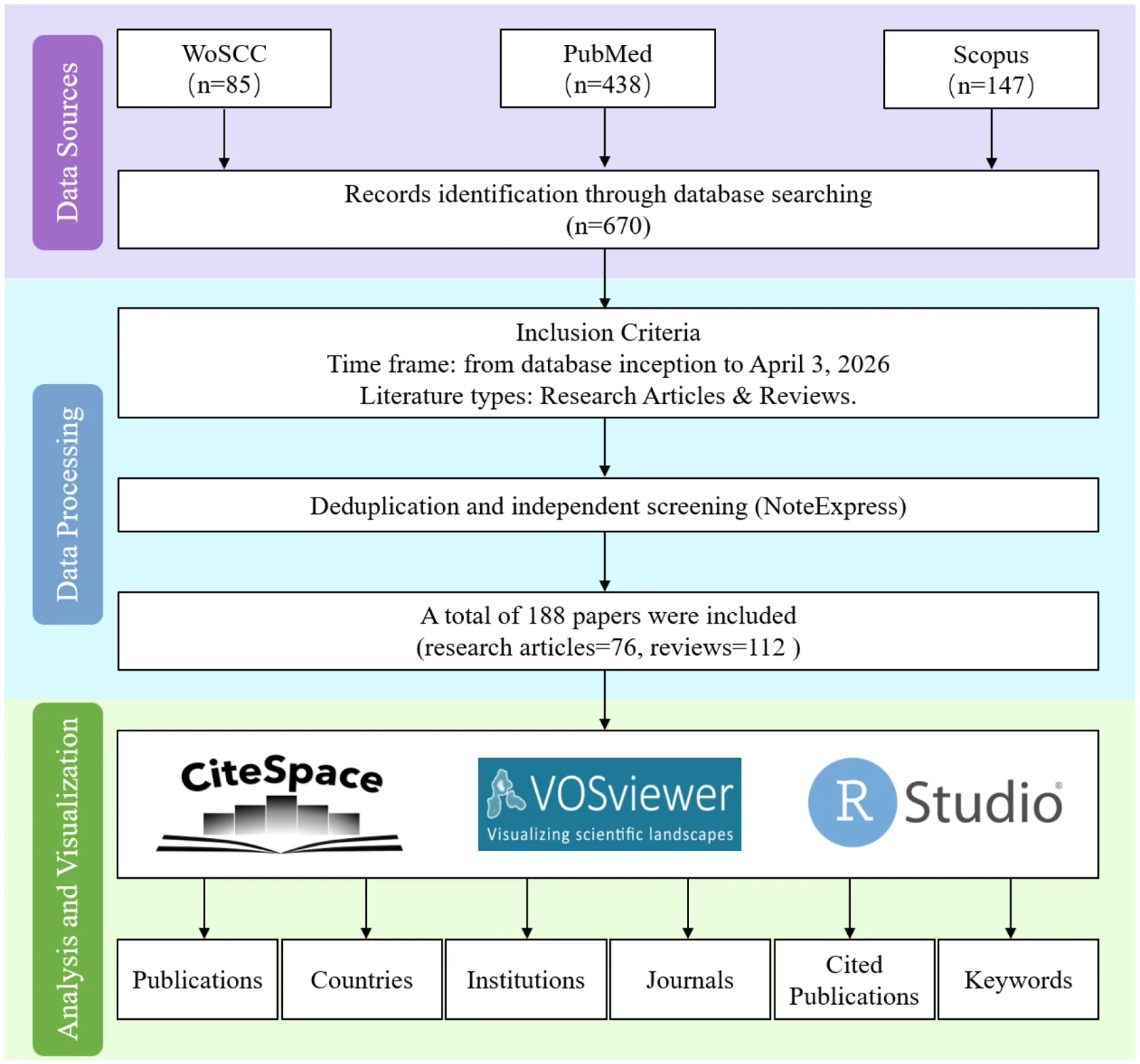
The research framework and workflow.

The overall research framework of this bibliometric analysis is summarized in **Figure 1**. Data were retrieved from three major databases, the Web of Science Core Collection (WoSCC), PubMed, and Scopus, and processed according to predefined criteria. Visualization analysis and trend exploration were performed using VOSviewer (1.6.20), CiteSpace (6.4.R1), and the R-based Bibliometrix package (4.5.2). In addition, Microsoft Excel 2019, Charticulator, and Scimago Graphica (1.0.53) were used for data organization and visualization.

### 2.1 Data sources and search strategy

Data were retrieved from three major databases: WoSCC, PubMed, and Scopus. These three are widely used in bibliometric studies for their broad coverage of peer-reviewed research in the life sciences and medicine. The combination also balances comprehensive scope with the data homogeneity required for software-based analysis (Chen et al., 2026).

The search queries varied across databases. In WoSCC, the query was: TS = ((exercise OR “physical activity” OR sports OR training) AND (exosome* OR “extracellular vesicle*” OR microvesicle*) AND (“non-coding RNA” OR ncRNA OR miRNA OR microRNA* OR lncRNA OR “long non-coding RNA” OR circRNA OR “circular RNA”) AND (cardiovascular OR heart OR cardiac OR myocardial OR vascular OR endothelium OR cardioprotection)). In PubMed, the query used was: ((“Exercise”[Mesh] OR “Physical Conditioning, Human”[Mesh] OR “Sports”[Mesh] OR “Physical Fitness”[Mesh] OR “Physical Exertion”[Mesh] OR exercise[tiab] OR “physical activity”[tiab] OR sports[tiab] OR training[tiab]) AND (“Exosomes”[Mesh] OR “Extracellular Vesicles”[Mesh] OR “Cell-Derived Microparticles”[Mesh] OR exosome*[tiab] OR “extracellular vesicle*”[tiab] OR microvesicle*[tiab]) AND (“RNA, Untranslated”[Mesh] OR “MicroRNAs”[Mesh] OR “RNA, Long Noncoding”[Mesh] OR “RNA, Circular”[Mesh] OR “non-coding RNA”[tiab] OR ncRNA[tiab] OR miRNA[tiab] OR microRNA*[tiab] OR lncRNA[tiab] OR “long non-coding RNA”[tiab] OR circRNA[tiab] OR “circular RNA”[tiab]) AND (“Cardiovascular Diseases”[Mesh] OR “Cardiovascular System”[Mesh] OR “Heart”[Mesh] OR “Myocardium”[Mesh] OR “Endothelium”[Mesh] OR “Vascular Diseases”[Mesh] OR cardiovascular[tiab] OR heart[tiab] OR cardiac[tiab] OR myocardial[tiab] OR vascular[tiab] OR endothelium[tiab] OR cardioprotection[tiab])). In Scopus, the query used was: TITLE-ABS-KEY ((exercise OR “physical activity” OR sports OR training) AND (exosome* OR “extracellular vesicle*” OR microvesicle*) AND (“non-coding RNA” OR ncRNA OR miRNA OR microRNA* OR lncRNA OR “long non-coding RNA” OR circRNA OR “circular RNA”) AND (cardiovascular OR heart OR cardiac OR myocardial OR vascular OR endothelium OR cardioprotection)). The search covered publications from database inception to April 3, 2026.

### 2.2 Data extraction, cleansing, and standardization

**Figure 2:**
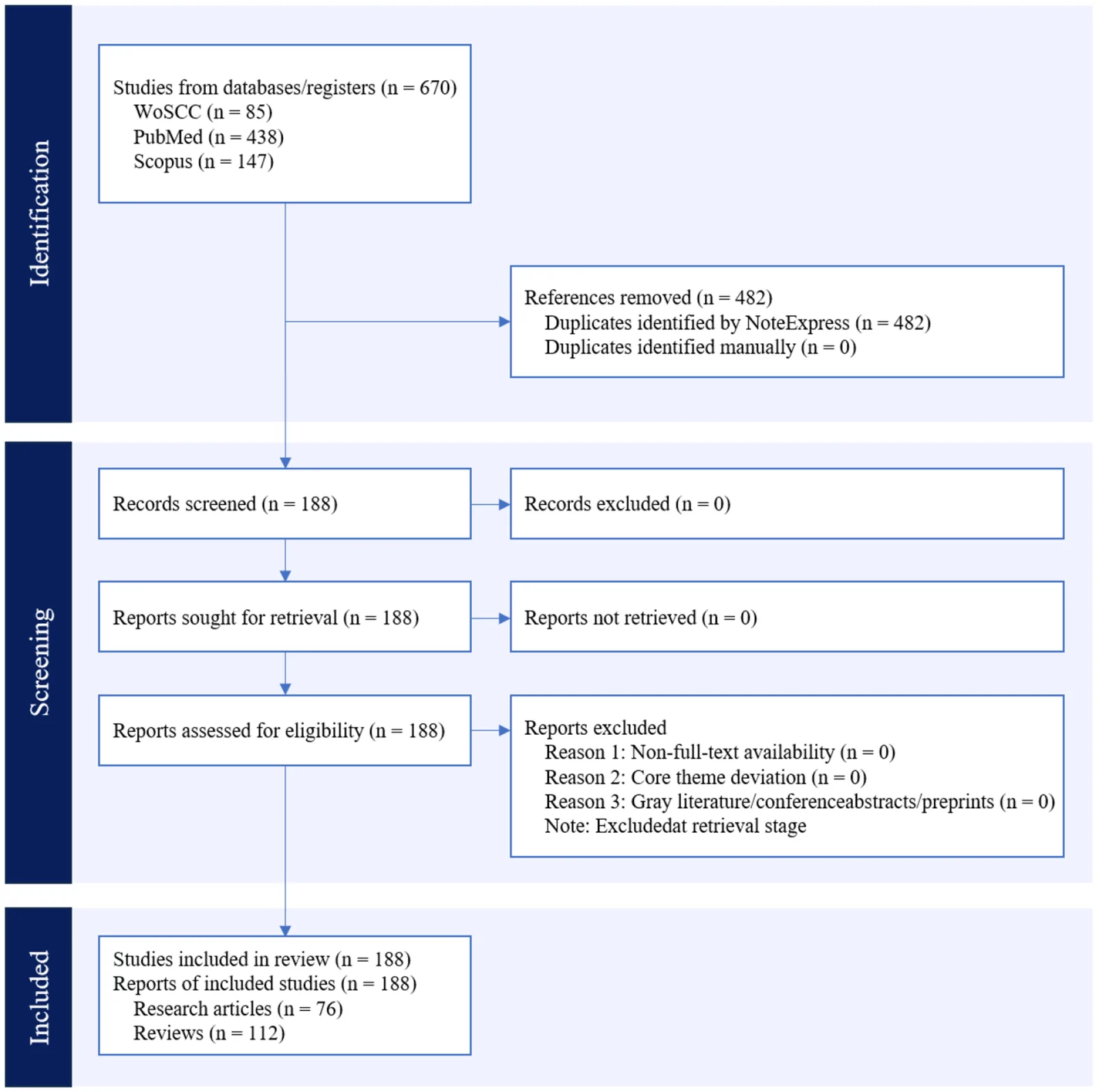
PRISMA flow diagram of literature screening and selection.

We used a PRISMA 2020-style flow diagram to clarify the screening workflow (**Figure 2**). Two reviewers independently screened the records for relevance to the study objectives to minimize subjective bias and ensure reliability. Disagreements were resolved by introducing a third reviewer and discussing until consensus was reached. For duplicate removal, we used NoteExpress to detect and remove duplicates, first by automatic comparison of titles, authors, and publication years, followed by manual verification of uncertain cases. During screening, we excluded non-English articles, duplicates, publications with incomplete bibliographic information, topic-irrelevant studies, and non-academic items (e.g., editorials, news, letters, conference abstracts). We included only original research articles and reviews. For inclusion, we selected studies that focused on exercise-mediated EV-derived ncRNAs in cardiovascular contexts, covering all ncRNA subtypes and diverse cardiovascular outcomes.

To harmonize records across databases, we manually merged and standardized entries for countries, institutions, and keywords to ensure consistency. For journals, we unified variant names under a single standard form. To ensure methodological consistency and reproducibility, we adopted equivalent search strategies with consistent logical frameworks across all three databases. To minimize potential database bias, we selected three major databases with different and complementary coverage scopes: WoSCC for standardized citation data, PubMed for comprehensive biomedical coverage, and Scopus for interdisciplinary breadth. The initial database search of WoSCC, PubMed, and Scopus retrieved 85, 438, and 147 records respectively, summing to a total of 670 records. After deduplication and screening, the total pool was narrowed to 188 eligible papers, comprising 76 research articles and 112 reviews.

In this manuscript, we consistently use “EV-derived” for our own interpretations and conclusions, following Minimal Information for Studies of Extracellular Vesicles 2023 guidelines. Two categories of source-derived nomenclature remain unmodified: (a) raw bibliometric keyword outputs extracted by analytical software; and (b) verbatim quoted material, including titles of cited publications. Retention of these historical “exosome/exosomal-related” terms reflects the source literature or software data and does not imply that we assign a specific biogenetic origin to the vesicles under investigation.

### 2.3 Visualization and statistical analysis

We used VOSviewer (1.6.20), CiteSpace (6.4.R1), and the R-based Bibliometrix package (4.5.2) for bibliometric analysis. VOSviewer constructed co-occurrence networks of countries and institutions. CiteSpace performed discipline analysis, keyword co-occurrence, clustering, and burst detection. Bibliometrix handled journal analysis, highly cited paper analysis, research trends, and three-field plots. Discrepancies between software outputs were checked against the original data. This integrated approach leverages the strengths of each tool to build a multi-dimensional framework for robust and comprehensive bibliometric insights (He et al., 2026). We also used Microsoft Excel 2019, Charticulator, and Scimago Graphica (1.0.53) for auxiliary data organization and visualization.

### 2.4 Parameter setting

VOSviewer: minimum keyword occurrence=5; fractional counting.

CiteSpace: time slicing=1 year; burst detection=default; Selection Criteria: g-index (k=25), LRF = 3.0, L/N=10, LBY = 5, e=1.0.

Bibliometrix: automatic layout was adopted to optimize node distribution, and the Walktrap algorithm was used to identify thematic clusters with high internal connectivity.

### 2.5 Ethics

This study is a bibliometric analysis based solely on publicly available literature data and does not involve human or animal subjects; therefore, ethical approval was not required.

## 3 Results

### 3.1 Annual publication trends

**Figure 3:**
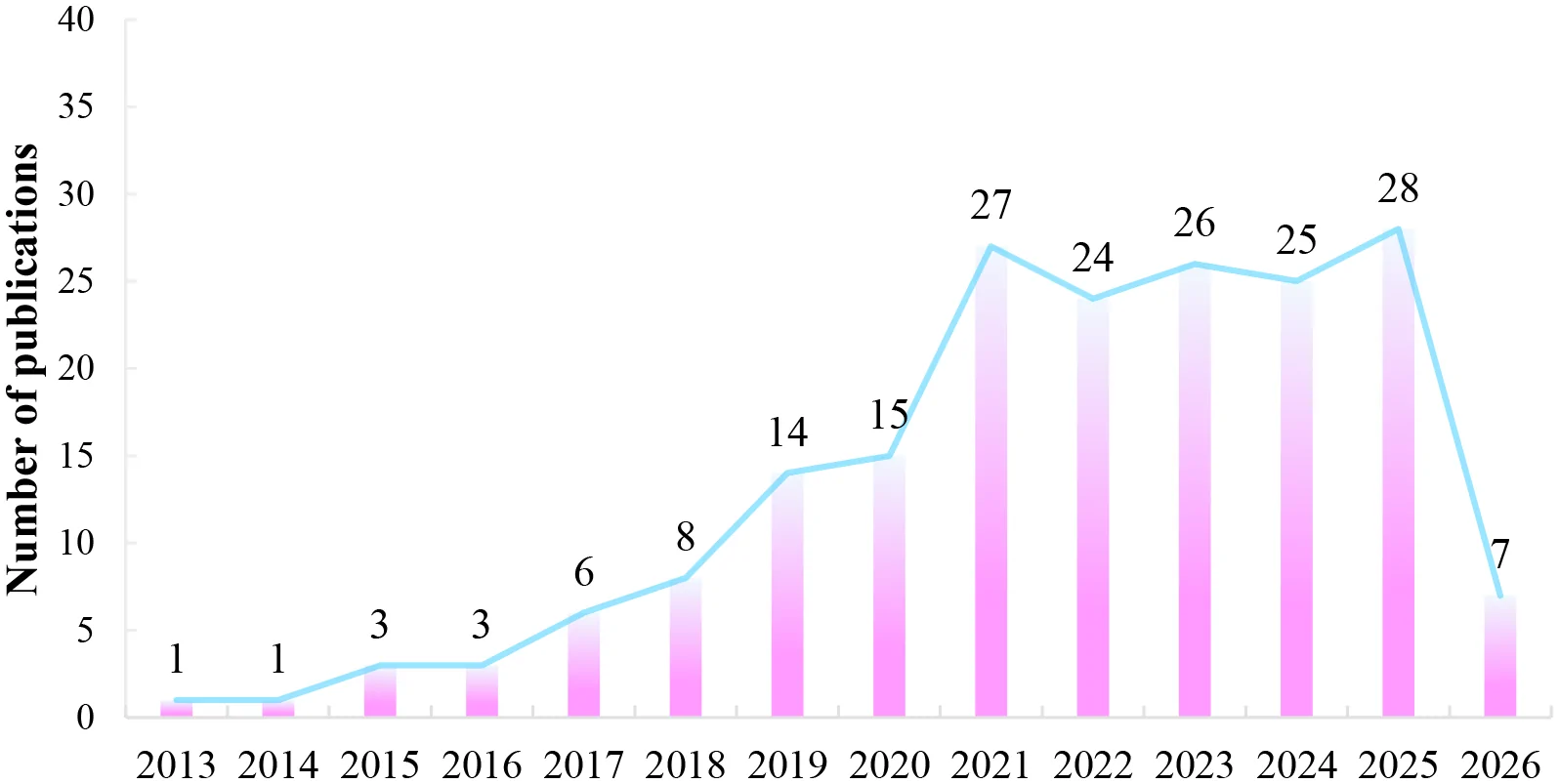
Annual volume of literature.

The annual publication count reflects the research activity and developmental trajectory of this field, rising from one publication in 2013 to 28 in 2025, with the first peak of 27 articles occurring in 2021. Between 2022 and 2025, annual output remained at a consistently high level of 24–28 publications (**Figure 3**). These trends suggest that as EV isolation and characterization techniques mature, and as ncRNA regulatory networks are progressively elucidated, exercise-mediated EVs are gaining sustained attention as key carriers in cardiovascular protection.

### 3.2 Country analysis

**Figure 4:**
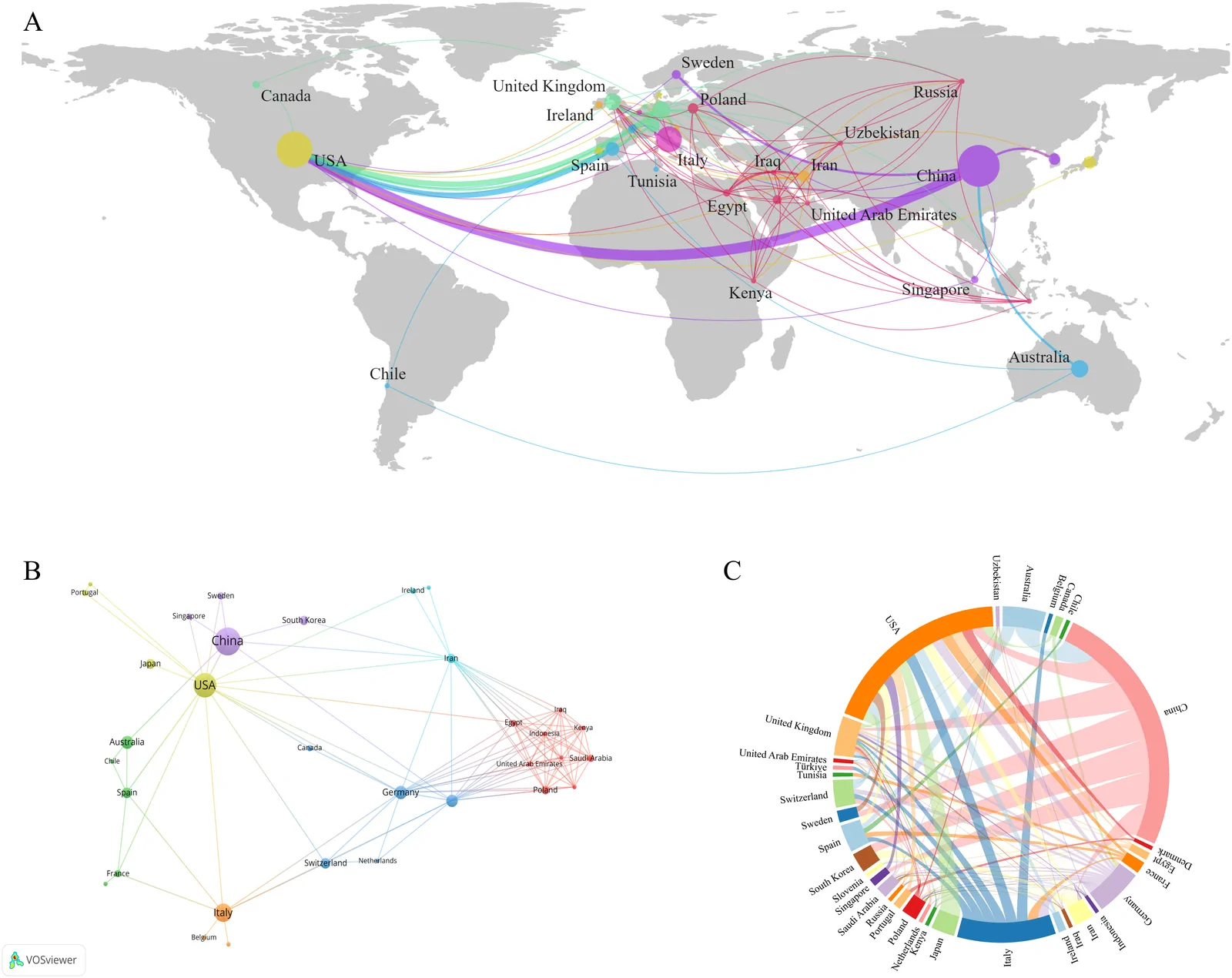
Co-occurrence map of countries. **(A)** Global distribution of scientific output and international co-authorship links. **(B)** Network visualization of countries collaborations. **(C)** Network map visualizing co-authorship relationships among countries.

**Figure 4A** shows the geographic distribution of the publications. The 33 contributing countries span North America, Europe, Asia, and Oceania, reflecting a globally distributed research landscape. As shown in **Figure 4B** and **Table 1**, research output is concentrated in China (65 publications) and the United States (48 publications). China leads the field, with a strong focus on exercise-mediated EV-derived ncRNAs and cardiovascular protection. **Figure 4C** illustrates the international collaboration network. China ranks first in volume and maintains close ties with the United States, the United Kingdom, and Sweden. These connections underscore its growing integration into the global research community.

**Table 1:** Top ten countries of publication volume.

Rank	Country	Counts
1	China	65
2	USA	48
3	Italy	25
4	Germany	12
5	Australia	11
6	United Kingdom	10
7	Spain	7
8	Switzerland	7
9	Japan	6
10	Iran	5

### 3.3 Institution analysis

**Figure 5:**
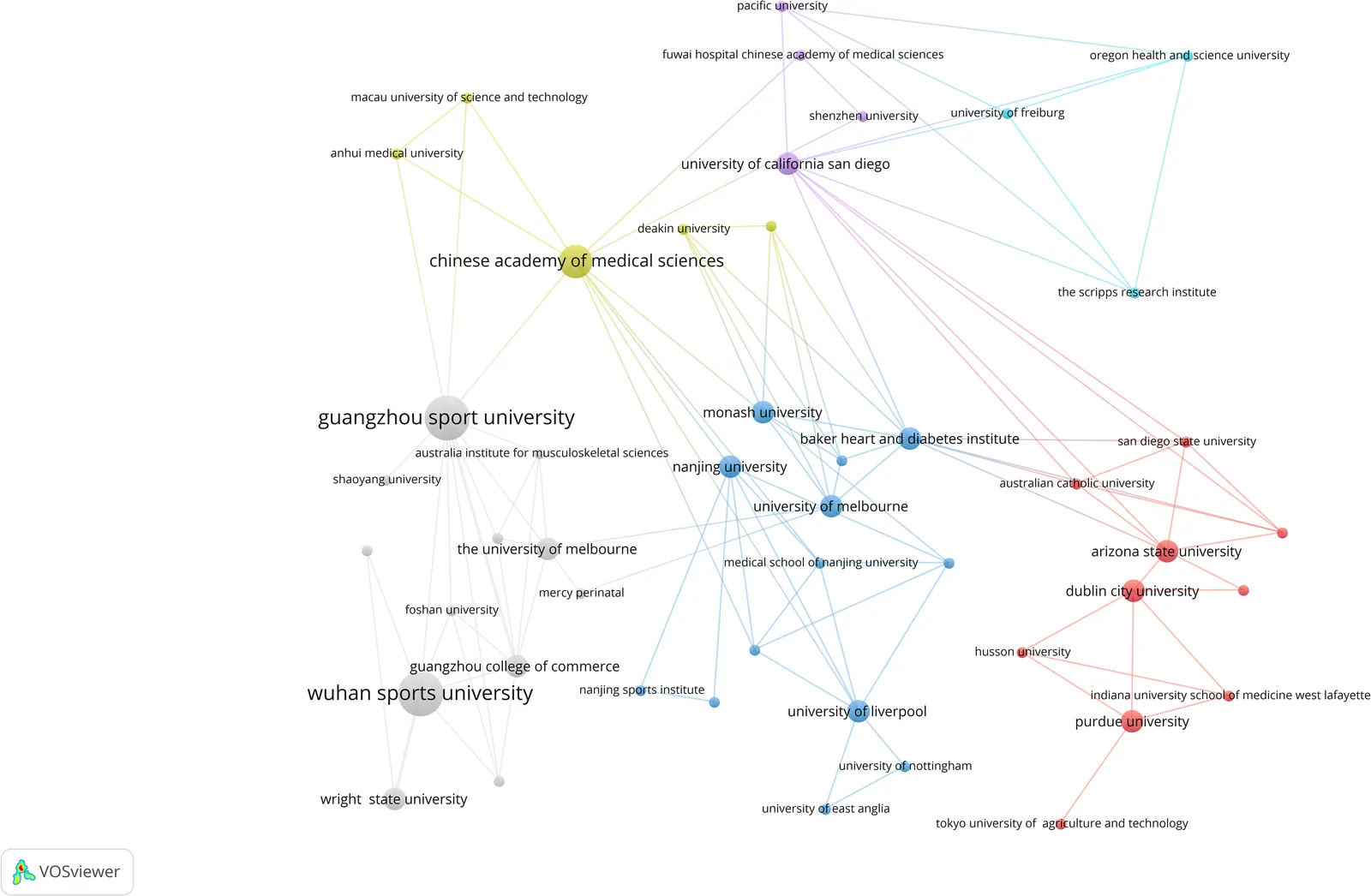
Institutional collaboration network map.

**Figure 5** and **Table 2** highlight the contributions of several institutions. The University of Zurich, Guangzhou Sport University, Wuhan Sports University, and the Fourth Military Medical University each published four articles. Over half of the institutions with three or more publications are from China, suggesting a strong concentration of core research capacity. These institutions span sport universities, comprehensive medical schools, and military medical institutes, reflecting a diverse and synergistic research landscape. Universities (12 institutions) are the primary research force, with research institutes (4 institutions) serving as supplements. This sustained investment has advanced fundamental research and facilitated exploration of clinical applications and novel therapeutic strategies.

**Table 2:** Institutions with three or more publications.

Rank	Institution	Country	Counts
1	University of Zurich	Switzerland	4
2	Guangzhou Sport University	China	4
3	Wuhan Sports University	China	4
4	Fourth Military Medical University	China	4
5	Instituto de Salud Carlos III	Spain	3
6	German Center for Cardiovascular Research	Germany	3
7	Università della Svizzera italiana	Switzerland	3
8	Chinese Academy of Medical Sciences	China	3
9	Karolinska Institutet	Sweden	3
10	Shanghai University	China	3
11	Sapienza University of Rome	Italy	3
12	University of Verona	Italy	3
13	Tongji University School of Medicine	China	3
14	Baltimore Veterans Affairs Geriatric Research	USA	3
15	Sichuan University	China	3
16	Central South University	China	3

### 3.4 Journal and discipline analysis

**Figure 6:**
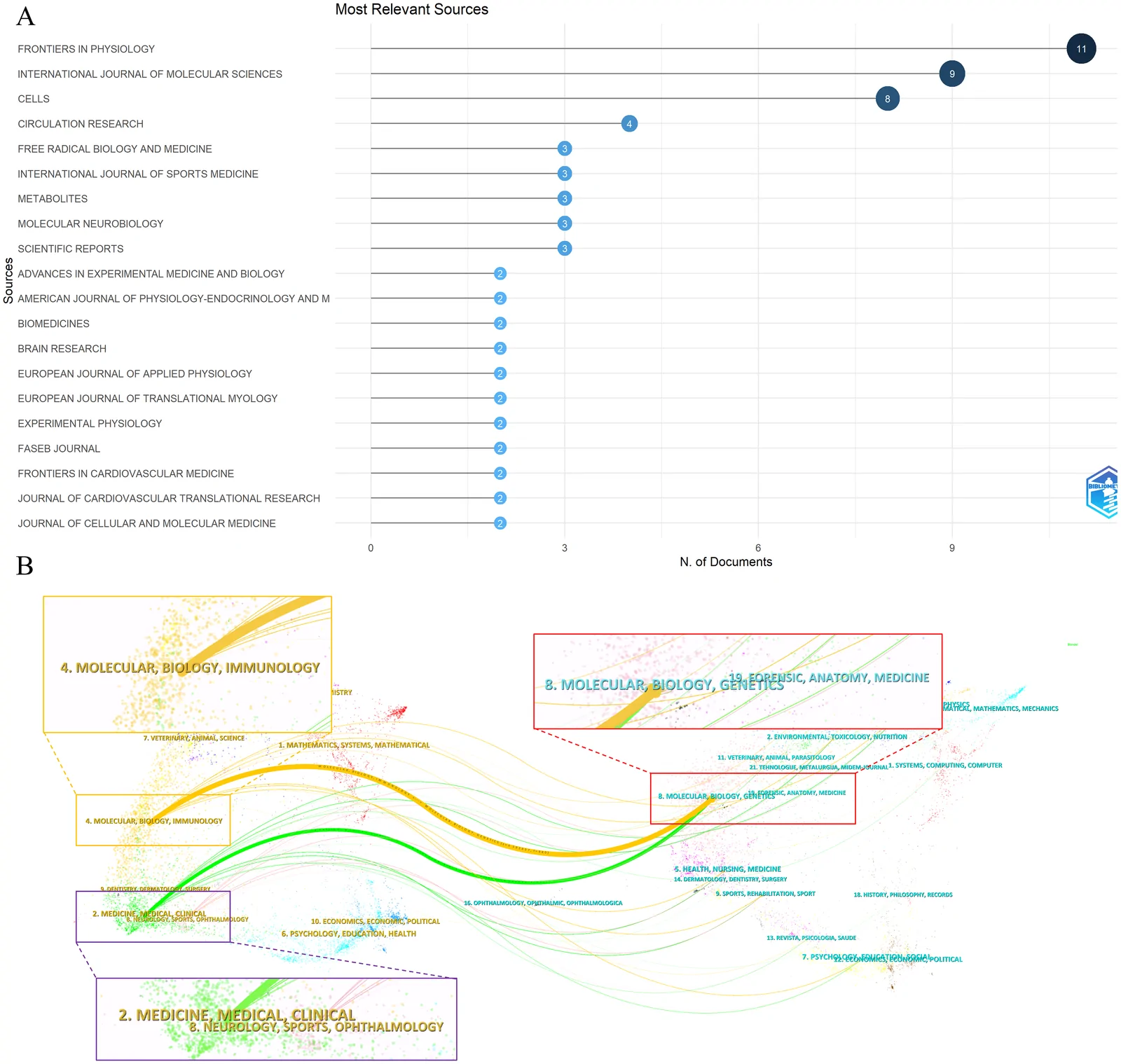
Analysis of journals. **(A)** The 20 most relevant sources. **(B)** Dual-map overlay analysis of interdisciplinary knowledge flow.

The included publications appear in 133 journals. Frontiers in Physiology leads with 11 publications (**Figure 6A**, **Table 3**), followed by the International Journal of Molecular Sciences (9 publications) and Cells (8 publications). Among journals with three or more publications, 77.78% were classified as Q1. Related work has also appeared in high-impact journals such as Signal Transduction and Targeted Therapy and Nature Reviews Cardiology, indicating growing interest from leading journals. The journal dual-map overlay (**Figure 6B**) reveals that citing journals are mainly in Molecular/Biology/Immunology and Medicine/Medical/Clinical, whereas the cited journals fall into Molecular/Biology/Genetics. This suggests a citation flow from basic and translational research toward clinical disciplines.

**Table 3:** Journals with three or more publications.

Rank	Journals	Counts	IF	JCR
1	Frontiers in Physiology	11	3.4	Q1
2	International Journal of Molecular Sciences	9	3.4	Q1
3	Cells	8	4.9	Q1
4	Circulation Research	4	16.2	Q1
5	Free Radical Biology and Medicine	3	8.2	Q1
6	International Journal of Sports Medicine	3	2.2	Q2
7	Metabolites	3	3.7	Q2
8	Molecular Neurobiology	3	4.3	Q1
9	Scientific Reports	3	3.9	Q1

### 3.5 Highly cited publication analysis

**Figure 7:**
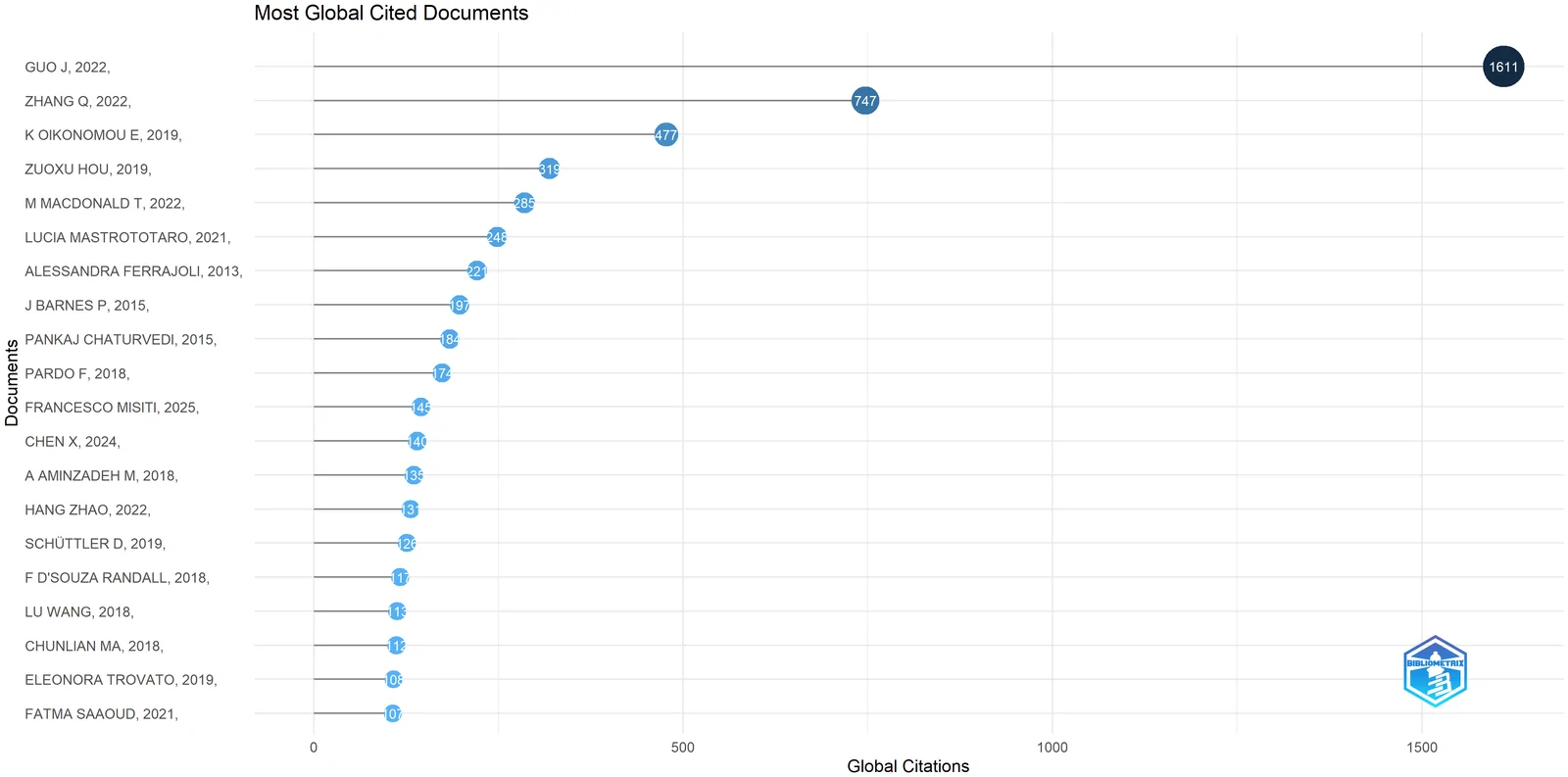
The 20 most globally cited documents.

**Figure 7** and **Table 4** present the highly cited publications in this field. Seven publications focus on signaling pathways, molecular mechanisms, and therapeutic strategies in cardiovascular and metabolic diseases (#1, #2, #3, #5, #6, #7, #8). The other three publications address the direct mechanisms of exercise-mediated EVs in cardiovascular protection (#4, #9, #10). A review in Signal Transduction and Targeted Therapy tops the list (1,611 citations). It systematically describes the molecular mechanisms and intervention strategies for aging and related diseases (Guo et al., 2022). The most cited original article is a study in Circulation Research (319 citations). It was the first to report that circulating EVs from long-term exercise confer cardiac protection via miR-342-5p, supporting the notion that cargo carried by exercise-mediated EVs can function as a novel class of exerkines (Hou et al., 2019).

**Table 4:** The top 10 cited articles.

Rank	Paper title	Journal	Citations	Year
1	Aging and aging-related diseases: from molecular mechanisms to interventions and treatments	Signal Transduction and Targeted Therapy	1611	2022
2	Signaling pathways and targeted therapy for myocardial infarction	Signal Transduction and Targeted Therapy	747	2022
3	The role of adipose tissue in cardiovascular health and disease	Nature Reviews Cardiology	477	2019
4	Longterm exercise-derived exosomal miR-342-5p: a novel exerkine for cardioprotection	Circulation Research	319	2019
5	Clinical tools and biomarkers to predict preeclampsia	eBioMedicine	285	2022
6	Insulin resistance and insulin sensitizing agents	Metabolism	248	2021
7	Prognostic value of miR-155 in individuals with monoclonal B-cell lymphocytosis and patients with B chronic lymphocytic leukemia	Blood	221	2013
8	Mechanisms of development of multimorbidity in the elderly	European Respiratory Journal	197	2015
9	Cardiosome mediated regulation of MMP9 in diabetic heart: role of mir29b and mir455 in exercise	Journal of Cellular and Molecular Medicine	184	2015
10	Extracellular vesicles in obesity and diabetes mellitus	Molecular Aspects of Medicine	174	2018

### 3.6 Keyword analysis

Keyword co-occurrence analysis assesses the frequency of keyword pairs in publications. The resulting patterns reveal thematic linkages and research priorities. High-frequency keywords mark core research directions. Keywords with high centrality connect disparate topics, underscoring their interdisciplinary relevance (Chen et al., 2026).

**Figure 8:**
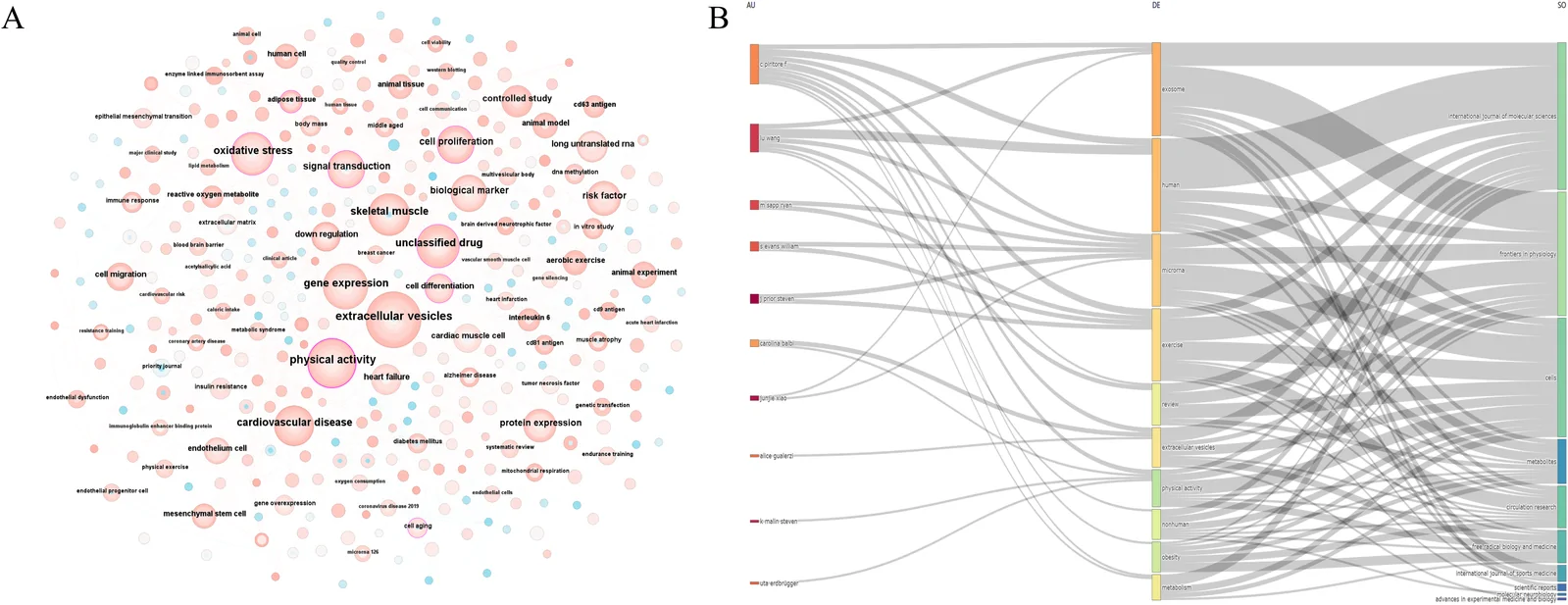
Keywords in exercise-mediated EV-derived ncRNAs and cardiovascular protection. **(A)** Keyword co-occurrence map. **(B)** The tripartite network visualization of “Author-keyword-journal”.

**Figure 8A** shows the keyword co-occurrence network. High-frequency terms mark research hotspots. High-centrality terms identify key topics. **Table 5** ranks the top 10 keywords by frequency and centrality. “Extracellular vesicles” appears most often (48 times), while “physical activity” has the highest centrality (0.22).

**Table 5:** The top 10 keywords by frequency and centrality.

Rank	Keywords	Frequency	Rank	Keywords	Centrality
1	extracellular vesicles	48	1	physical activity	0.22
2	physical activity	36	2	oxidative stress	0.18
3	gene expression	33	3	unclassified drug	0.17
4	unclassified drug	29	4	signal transduction	0.15
5	cardiovascular disease	28	5	cell proliferation	0.12
6	oxidative stress	27	6	cell aging	0.12
7	skeletal muscle	27	7	cell differentiation	0.11
8	biological marker	22	8	adipose tissue	0.1
9	cell proliferation	22	9	gene expression	0.08
10	signal transduction	21	10	cardiovascular disease	0.07

The tripartite network mapped bibliometric relationships as interconnected node-edge structures (**Figure 8B**) (Wang et al., 2025). The author–keyword co-occurrence linked Professors Francesca Cristiana Piritore and Lu Wang to the “human” and “exosome” keyword clusters, confirming their interest in EV functions in human physiology and pathology. The journal–keyword analysis revealed distinct patterns. “Exosome” articles clustered in Frontiers in Physiology, “human” articles in International Journal of Molecular Sciences, and “miRNA” articles in Cells. This network topology analysis identifies the core scholars and their research directions in the field, while also establishing Frontiers in Physiology, the International Journal of Molecular Sciences, and Cells as important platforms for disseminating research in this discipline.

**Figure 9:**
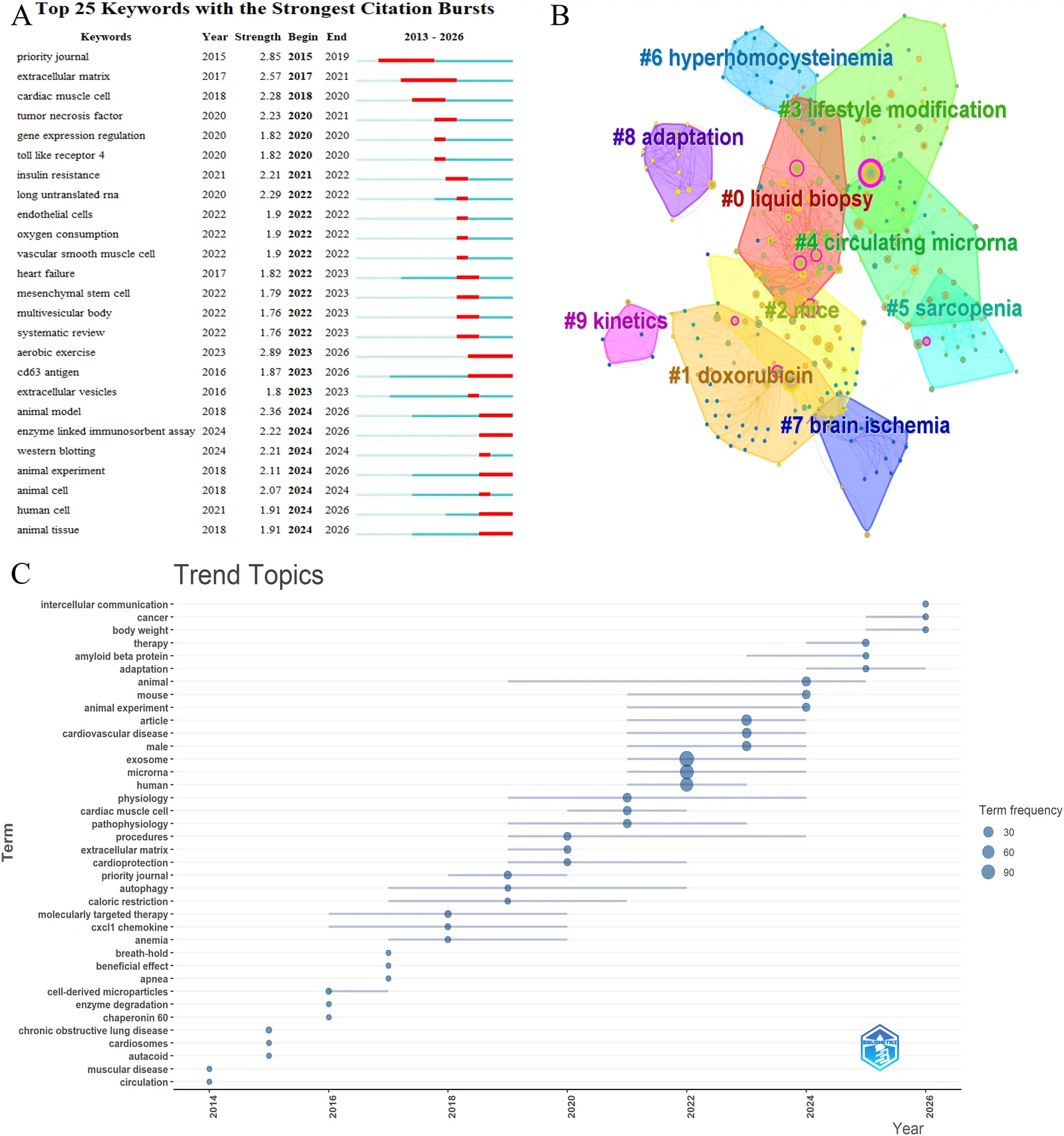
Keywords analysis of exercise-mediated EV-derived ncRNAs and cardiovascular protection. **(A)** Highlights the top 25 keywords with the strongest citation bursts. **(B)** Clustering map of keywords. **(C)** Trend topics.

**Figure 9A** tracks citation bursts for keywords from 2000 to 2024, highlighting the top 25. The bursts fall into three phases.

Phase 1 (2015–2020): early foundational exploration. In 2015, “priority journal” burst (intensity 2.85) and lasted until 2019, reflecting early work in basic medicine and clinical journals. In 2017, “extracellular matrix” burst (intensity 2.57) through 2021, indicating growing attention to matrix remodeling. In 2018, “cardiac muscle cell” (intensity 2.28) and “tumor necrosis factor” (intensity 2.23) appeared, shifting focus to cardiomyocyte injury and inflammation. In 2020, “gene expression regulation” and “toll like receptor 4” showed short bursts, suggesting a phase of scattered exploration.

Phase 2 (2021–2023): expansion and interdisciplinary integration. “Insulin resistance” (intensity 2.21) burst in 2021, and “long untranslated RNA” (intensity 2.29) in 2022, introducing metabolism and lncRNA topics. In 2022, multiple keywords including “endothelial cells,” “vascular smooth muscle cells,” “mesenchymal stem cells,” “heart failure,” and “multivesicular body” burst concurrently, expanding research dimensions from cardiomyocyte injury to systemic vascular pathology, heart failure and EV biogenesis. In 2023, “aerobic exercise” (intensity 2.89) burst and continues through 2026, establishing exercise intervention as a hotspot.

Phase 3 (2023–2026): frontier deepening. After 2023, “cd63 antigen” (intensity 1.87) and “extracellular vesicles” (intensity 1.8) burst sequentially. As a canonical marker of EVs, lysosomal-associated membrane protein 3, alongside the widespread co-occurrence of “extracellular vesicles,” identifies EVs as a fast-growing research hotspot. In 2024, multiple keywords burst simultaneously, including “animal model,” “animal experiment,” “animal cell,” “animal tissue,” and “human cell.” These keywords remain prominent through 2026, suggesting a shift from *in vitro* and animal models toward human cell validation. Concurrently, technical keywords such as “enzyme linked immunosorbent assay” (intensity 2.22) and “western blotting” (intensity 2.21) also burst, suggesting a move from phenomenological description to mechanistic validation.

Recent burst keywords reveal a shift from disease mechanisms toward an exercise–EV–human validation axis. This reflects deeper integration of translational and mechanistic research.

**Figure 9B** and **Table 6** present the keyword clustering results, covering EV biology, cardiovascular pathology, exercise intervention, and experimental techniques. Cluster #0 covers EV-mediated intercellular communication, vascular remodeling, myocardial infarction, and mesenchymal stem cells. Cluster #1 includes myocardial infarction, signal transduction, epithelial–mesenchymal transition, oxidative phosphorylation, and vascular remodeling. Cluster #2 involves skeletal muscle, physical activity, and runt-related transcription factor 2. Cluster #3 covers diabetes, cardiovascular risk, sodium–glucose cotransporters, and EVs as a key link. Cluster #4 includes EVs, circulating miRNAs, kidney injury, muscle atrophy, and skeletal muscle. Cluster #5 covers resistance training, myocardial infarction, and Ras homolog family member A guanosine triphosphatase. It suggests exercise intervention’s value in sarcopenia and CVD. Cluster #6 involves diabetes, ischemic preconditioning, endoplasmic reticulum stress, and reactive oxygen metabolites. Cluster #7 covers mechanistic target of rapamycin signaling, endothelial cells, and endothelial dysfunction. Cluster #8 includes collagen types, peripheral arterial disease, signal transduction, runt-related transcription factor 2, and tissue adaptive remodeling. Cluster #9 integrates energy metabolism, signal transduction, exercise, hypoxia, shear stress, inflammation, and miRNAs. It provides the most direct theoretical foundation for this study.

**Table 6:** Keyword clustering table.

Cluster ID	Silhouette	Mean (Year)	Label (LSI)
#0 liquid biopsy	0.755	2020	binding protein; heart infarction; sonic hedgehog protein; ischemic preconditioning; stimulating factor; extracellular vesicles; cell communication; vascular remodeling; mesenchymal stem cell; multiple myeloma
#1 doxorubicin	0.888	2018	heart infarction; signal transduction; sonic hedgehog protein; epithelial-mesenchymal transition; oxidative phosphorylation; cell communication; vascular remodeling; mesenchymal stem cell; epidermal growth factor receptor; particle size
#2 mice	0.9	2020	skeletal muscle; physical activity; *in vivo* study; cell communication; biological marker; human umbilical vein; coronavirus disease; transcription factor RUNX2; umbilical vein; endothelial cells
#3 lifestyle modification	0.779	2021	diabetes mellitus; dependent diabetes mellitus; cardiovascular risk; sodium-glucose cotransporter; peripheral occlusive artery disease; extracellular vesicles; artificial intelligence; delayed graft function; graft survival; graft rejection
#4 circulating miRNA	0.704	2021	extracellular vesicles; collagen type; kidney injury; muscle atrophy; skeletal muscle; circulating miRNA; analytical phase; circulating miRNA; gene function; endocrine system
#5 sarcopenia	0.935	2020	neurotrophic factor; histone deacetylase; alzheimer disease; blocking agent; binding affinity; resistance training; heart infarction; binding protein; sonic hedgehog protein; RhoA GTPase
#6 hyperhomocysteinemia	0.956	2018	sodium-glucose cotransporter; diabetes mellitus; dependent diabetes mellitus; kidney disease; risk factor; ischemic preconditioning; endoplasmic reticulum stress; reactive oxygen metabolite; mesenchymal stem cell; diabetic cardiomyopathy
#7 brain ischemia	0.85	2015	mammalian target of rapamycin; metabolic diseases; pulmonary disease; preventive medicine; cardiovascular diseases; endothelial cells; endothelial dysfunction; vascular disease
#8 adaptation	0.959	2024	collagen type; peripheral arterial disease; growth factor; thrombocyte activation; limb ischemia; signal transduction; transcription factor RUNX2; mesenchymal stem cell; coronavirus disease
#9 kinetics	0.99	2015	energy metabolism; signal transduction; gene expression regulation; inflammation; inflammation mediators; exercise; hypoxia; shear stress; C-reactive protein; miRNA

From a temporal perspective, early research (Clusters #7 and #9) focused on energy metabolism and endothelial dysfunction. The middle phase (Clusters #0, #1, #2, #5, and #6) expanded toward EVs and signal transduction. Recent directions (Clusters #3, #4, and #8) have concentrated on lifestyle interventions, circulating miRNAs, and tissue adaptation. These clusters are interconnected through shared keywords, including EVs, signal transduction, and mesenchymal stem cells. Together, they form the research network in this field.

Keyword timezone analysis reveals the evolution of research hotspots and their periodic shifts (**Figure 9C**). Early studies focused on the circulatory system and muscle diseases. High-frequency keywords included “circulation,” “muscular disease,” “autacoid,” “cardiosomes,” and “cell-derived microparticles.” The themes were relatively diffuse, spanning autophagy, targeted therapy, and caloric restriction. In the middle phase, keywords related to metabolism and EVs emerged. “Cardiovascular disease,” “cardioprotection,” “exosome,” and “miRNA” emerged as prominent terms, suggesting a deeper focus on cardiovascular protection mechanisms. In the most recent phase, keywords related to animal experiments, such as “animal experiment,” “mouse,” and “animal,” reappeared, marking a shift back toward fundamental biology.

## 4 Discussion

### 4.1 General information and novelty of the study

This study used VOSviewer, CiteSpace, and the R-based Bibliometrix package to map the research landscape of exercise-mediated EV-derived ncRNAs in cardiovascular protection. It identified core themes and emerging frontiers. The Results suggest that China and the United States are the leading contributors. Annual publication output has grown rapidly since 2021, suggesting that this field represents a growth area in cardiovascular translational medicine (Khetarpal et al., 2025).

To our knowledge, this is the first bibliometric analysis to integrate exercise, EVs, ncRNAs, and cardiovascular protection into a single cross-disciplinary framework. Previous studies have addressed only one or two of these dimensions. We constructed a cross-disciplinary knowledge map covering all four areas. We also traced the field’s evolution from early observations to mechanistic and translational studies.

Exercise-mediated EV-derived miRNAs have been extensively investigated in preclinical models for their roles in cardiovascular protection. Seminal work reported that circulating EVs from long-term exercise training confer cardioprotection via miR-342-5p, positioning EV-derived miRNAs as core exerkines (Hou et al., 2019). Subsequent studies have extended these findings to other miRNAs, tissues, and disease contexts (Liu et al., 2025; Guo et al., 2025; Chen and Zhang, 2025). Together, these studies provide supporting context and background rationale for our bibliometric analysis.

We discuss three ways our findings relate to the literature. They align with, extend, and go beyond previous studies.

Alignment with existing literature at the level of research subjects. A growing body of evidence has suggested that exercise-mediated EV-derived ncRNAs contribute to cardiovascular protection via EV markers, miRNAs, and P-element-induced wimpy testis-interacting RNAs (Lai et al., 2023; Wang H. et al., 2026). Our keyword analysis identified “exosome” as a core keyword, along with “miRNA,” “exercise,” and “cardioprotection.” These themes are consistent with the cited literature.

Extension beyond existing literature in scope. Existing bibliometric studies have focused either on EVs in specific diseases or on exercise in cardiovascular health. They have not systematically examined the relationship among exercise, EVs, ncRNAs, and cardiovascular protection. We expanded the focus from single domains to an integrated framework covering all four topics. We also built a tripartite author–keyword–journal network. This extends previous bibliometric approaches beyond citation and keyword analyses to include collaborative and structural perspectives.

Methodological advancement beyond existing literature. We refined the interpretation of algorithm-generated cluster labels. We did not adopt them at face value but re-examined the clusters. Clusters #7 and #9 provided a theoretical foundation for early development. Cluster #5 reflected multi-system crosstalk in exercise intervention. These interpretations came from a content-based reading. Keyword burst detection showed a shift from disease mechanisms toward an exercise-EV-human validation axis. This trend has not been reported systematically before.

This study mapped the research landscape of exercise-mediated EV-derived ncRNAs in cardiovascular protection. Using a multidimensional framework, it revealed the knowledge structure, evolutionary patterns, and frontier trends. These findings highlight potential research fronts that may aid future translational investigations.

### 4.2 Mechanistic insights

We combined our bibliometric outputs with available published experimental evidence to outline potential mechanistic pathways. These pathways represent our interpretive synthesis of the literature, not direct statistical outputs from our bibliometric analysis. These pathways were selected based on several observations from our bibliometric analysis. “Oxidative stress” showed high centrality. “Signal transduction” co-occurred across clusters. Keywords for cell injury and inflammation burst sequentially. “Extracellular matrix” and a tissue-remodeling cluster showed sustained bursts. Current evidence suggests that exercise-mediated EV-derived ncRNAs may influence several protective processes, including anti-apoptotic, antioxidant, anti-inflammatory, and anti-fibrotic effects, although whether these effects converge into a coordinated network *in vivo* remains to be established and warrants further investigation.

#### 4.2.1 Anti-apoptotic mechanisms

In myocardial ischemia/reperfusion injury, apoptosis contributes to cardiomyocyte death and cardiac dysfunction. Previous studies suggest that exercise may regulate cardiomyocyte apoptosis via EV-derived ncRNAs. Exercise may alter ncRNA expression in circulation and EVs, which may influence tissue pathology through cell proliferation, apoptosis, and angiogenesis (Wang Z. et al., 2026; Alshahrani et al., 2026). Preclinical evidence suggests that exercise-mediated EV-derived miRNAs may mitigate chronic heart failure, potentially through suppression of fibrosis, preservation of mitochondrial function, and promotion of cardiomyocyte survival (Wu et al., 2025). Exercise-regulated miRNAs have been suggested to modulate pro-survival, angiogenic, and anti-fibrotic signaling via phosphatidylinositol 3-kinase (PI3K)/protein kinase B (AKT) and nuclear factor kappa-B (NF-κB) pathways (Li et al., 2026). Exercise has been suggested to regulate cardiomyocyte apoptosis via multiple miRNAs. It may suppress miR-206, which appears to upregulate heat shock protein 60 and reduce apoptosis. It may also elevate miR-486-5p, which has been suggested to attenuate mammalian sterile 20-like kinase 1 activity and reduce apoptosis (Pu et al., 2021). In addition, exercise may downregulate miR-1, which may lead to increased insulin-like growth factor-1 and its receptor, and thereby attenuate apoptosis (Jing et al., 2026).

These mechanistic findings suggest that the molecular pathways linking exercise, EV-derived ncRNAs, and cardiomyocyte apoptosis have been investigated in preclinical models, and these observations align with our clustering analysis that highlighted “signal transduction” as a recurring theme, though this concordance does not establish a causal relationship.

However, most findings are derived from rodent models of acute ischemia/reperfusion injury, and it remains uncertain whether similar anti-apoptotic pathways are operative in human cardiomyocytes under chronic exercise conditions (Quindry and Franklin, 2021).

#### 4.2.2 Antioxidant mechanisms

Oxidative stress is a major contributor to post-infarction tissue injury. Evidence suggests that exercise counteracts reactive oxygen species elevation, in part via redox modulation. This effect may be partly driven by exercise-mediated EV release. Under oxidative stress or exercise, EVs appear to alter their cargo and may exert protective effects, potentially through enhanced antioxidant activity and improved redox balance (Lisi et al., 2023). This observation aligns with our clustering results. Exercise-mediated circulating EVs may be generated through both endosomal sorting complex required for transport-dependent and -independent pathways. Their secretion is modulated by Ras-related proteins in brain and soluble N-ethylmaleimide-sensitive factor attachment protein receptors. Mechanical stress, calcium homeostasis, metabolic status, and neuroendocrine signals may also contribute to the regulation of EV release (Wang X. et al., 2026). Skeletal muscle-derived EVs have been suggested to protect cardiomyocytes by delivering functional molecules including miRNAs and proteins. Bone-derived EVs may improve CVD by modulating oxidative stress and inflammation (Li Q. et al., 2025). These findings are in line with clusters related to cell communication and skeletal muscle in our analysis.

A resistance training cluster suggests that exercise may benefit the skeletal muscle–cardiovascular interface. Resistance training has been shown to alter circulating EV profiles and their miRNA cargo (Csala et al., 2025). These EVs and their cargo could facilitate tissue adaptation following this form of exercise, and potentially extend the antioxidant mechanism from local myocardial protection to inter-organ communication. Exercise-mediated reactive oxygen species signaling may activate nuclear factor erythroid 2-related factor 2 (Nrf2) and to influence EV secretion, potentially mediating systemic health benefits (Louzada et al., 2020). Published evidence also suggests that these pathways may contribute to the regulation of mitochondrial biogenesis, antioxidant defense, and cytoprotection, though these associations remain to be causally established (Bouviere et al., 2021).

Furthermore, the relative contribution of EV-mediated versus EV-independent mechanisms to redox balance has yet to be quantified, and the lack of standardized ncRNA characterization methods across studies complicates direct data integration (Robinson et al., 2022).

#### 4.2.3 Anti-inflammatory mechanisms

Inflammation has a dual role in CVD progression. EV cargo includes miRNAs, proteins, and lipids, which play regulatory roles in immune responses with both pro- and anti-inflammatory functions. Evidence suggests that exercise may exert anti-inflammatory effects partly via changes in circulating EV composition and function (Catitti et al., 2022). This points to a possible EV-mediated mechanism for immune regulation. Exercise has been reported to downregulate miR-let-7c-5p in circulating EVs in some studies. This may upregulate tissue inhibitor of metalloproteinase 3 and reduce inflammatory factors such as tumor necrosis factor-α, interleukin-6, and matrix metalloproteinase-9 (MMP-9). These changes may help attenuate atherosclerosis and vascular inflammation in preclinical models (Guo et al., 2025). Exercise may also modulate NF-κB signaling, thereby regulating pro-inflammatory cytokines and indirectly influencing antioxidant defenses. In addition, exercise may stimulate EV release to mitigate inflammation. Together, these effects may contribute to systemic anti-inflammatory benefits (Ji, 2025; Li H. et al., 2025).

Nevertheless, current evidence for these anti-inflammatory effects is largely preclinical, underscoring the need for well-controlled human exercise trials to confirm their translational value (Lai et al., 2023).

#### 4.2.4 Anti-fibrotic mechanisms

Cardiac fibrosis is an important pathological feature of ventricular remodeling following myocardial infarction. Exercise may also attenuate cardiac fibrosis via EV-derived ncRNAs. Some studies have reported that exercise may upregulate miR-126 in endothelial progenitor cell-derived EVs. This has been associated with reduced cardiac fibrosis, possibly through transforming growth factor-β (TGF-β)/Sma- and mothers against decapentaplegic family member 3 (Smad3) inhibition (Fu et al., 2024). Exercise may also stimulate EV secretion from cardiac cells, including cardiomyocytes, fibroblasts, and endothelial cells. As demonstrated in preclinical work, among these EVs, miR-29b and miR-455 suppress MMP-9 expression by binding to the 3’ untranslated region of its mRNA, which could slow extracellular matrix degradation and fibrosis progression (Chaturvedi et al., 2015). Based on published evidence, resistance training may alter circulating EV-derived miRNA profiles. For instance, the miR-29 family may attenuate fibrosis, potentially through targeting extracellular matrix–related genes (Xhuti et al., 2023). Exercise-mediated skeletal muscle EVs may also contribute to this effect. High-intensity interval training has been reported to increase circulating EV-derived miR-133a levels by 2- to 3-fold. This increase may reduce cardiac fibrosis through targeting connective tissue growth factor (Yu et al., 2026).

The involvement of exercise-mediated EV-derived ncRNAs in inhibiting cardiac fibrosis has been suggested by a growing body of experimental evidence, primarily derived from preclinical models. These findings are in line with the keyword burst patterns in our analysis. Together, they may point to a molecular basis for the integrated cardiovascular protection mediated by exercise-mediated EV-derived ncRNAs.

Importantly, most fibrosis studies have used young healthy animals. Whether similar anti-fibrotic effects occur in aged or diseased populations remains unclear, yet this is where cardiac fibrosis is most clinically relevant. Clinical translation will likely require addressing challenges in EV manufacturing, delivery, and regulatory approval (Zhang J. et al., 2026).

#### 4.2.5 Multi-mechanism synergistic network

The four mechanisms may converge through shared signaling molecules and transcription factors. Although exercise-derived EVs are known to carry multiple miRNAs that individually affect distinct pathways, whether these ncRNAs operate in concert to produce synergistic effects remains a conceptual hypothesis rather than an empirically established mechanism (Huang et al., 2026). Exercise-mediated EVs may also deliver miRNAs, proteins, and lipids to non-contractile organs. This may coordinate multi-tissue responses that protect and repair the cardiovascular system (Perera et al., 2025). Available preclinical data suggest that certain EV-derived cargo molecules, including miR-342-5p, miR-126 and miR-29b, have been individually linked to the modulation of pathways such as PI3K/AKT, mitogen-activated protein kinase, NF-κB and TGF-β/Smad3 in various experimental settings (Wadley et al., 2019). We propose these cascades may converge to form a synergistic cardioprotective network; however, this remains a literature-informed conceptual framework and not a proven causal regulatory network. Thus, direct experimental validation is required to test the proposed interactions and their relative contributions *in vivo*, particularly in human models. It is further speculated that the exercise-related factor irisin may cooperate with exercise-induced EV cargo, although this hypothesis remains untested (Wang et al., 2024).

While these mechanistic interpretations may support a synergistic framework, several limitations warrant consideration. These include heterogeneity in EV isolation and ncRNA characterization, limited clinical evidence, and variability in experimental models. These limitations highlight the need for standardized protocols and large-scale human validation before clinical translation (Zhang et al., 2026).

**Figure 10:**
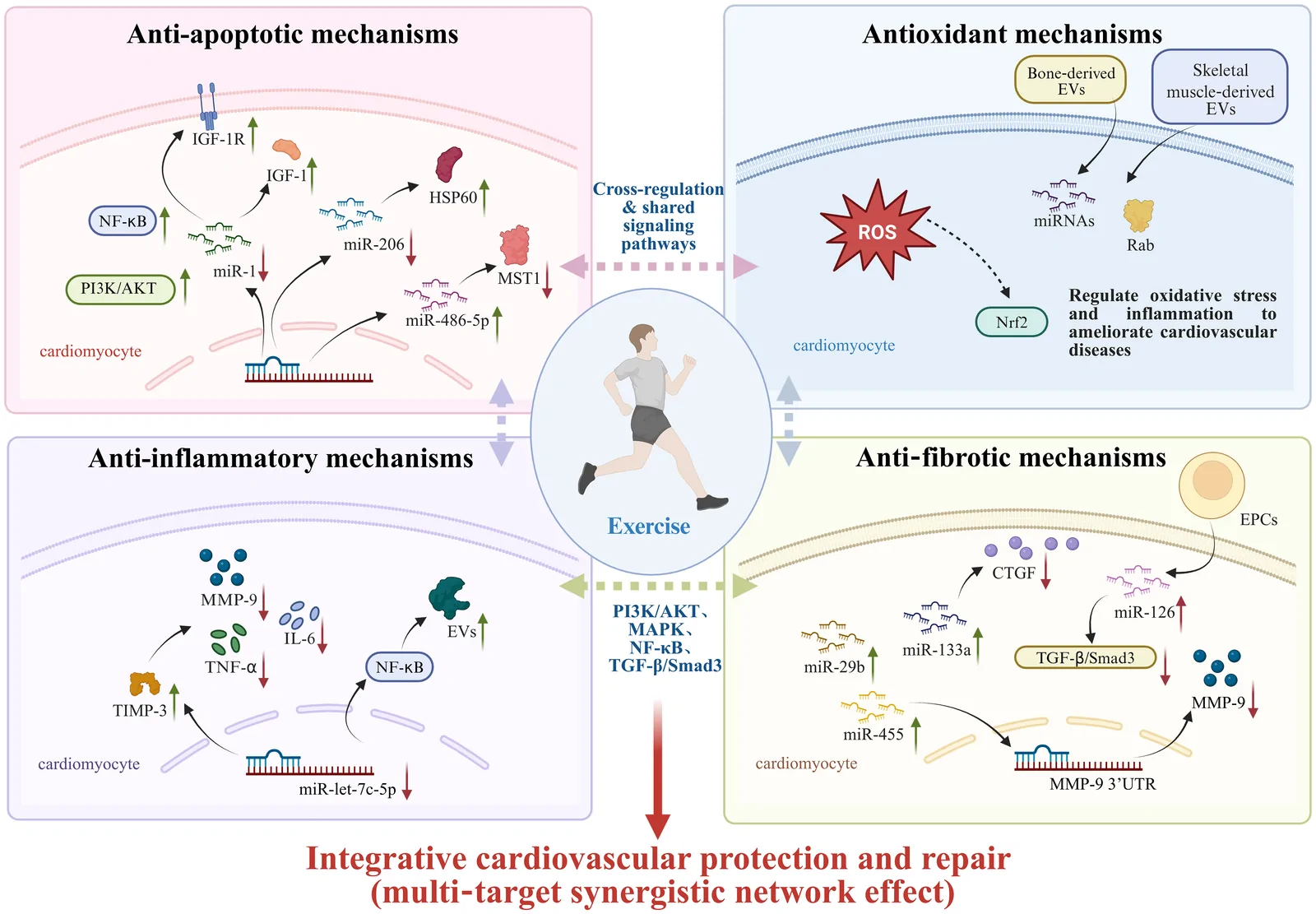
Conceptual schematic illustrating a literature-informed hypothetical framework for potential synergistic cardiovascular protective pathways associated with exercise-mediated EV-derived ncRNAs. Abbreviations: 3’UTR, 3’ untranslated region; AKT, protein kinase B; CTGF, connective tissue growth factor; EPCs, endothelial progenitor cells; EVs, extracellular vesicles; HSP60, heat shock protein 60; IGF-1, insulin-like growth factor-1; IGF-1R, insulin-like growth factor-1 receptor; IL-6, interleukin-6; MAPK, mitogen-activated protein kinase; MMP-9, matrix metalloproteinase-9; MST1, mammalian sterile 20-like kinase 1; NF-κB, nuclear factor kappa-B; Nrf2, nuclear factor erythroid 2-related factor 2; PI3K, phosphatidylinositol 3-kinase; Rab, Ras-related proteins in brain; ROS, reactive oxygen species; Smad3, Sma- and mothers against decapentaplegic family member 3; TGF-β, transforming growth factor-β; TIMP-3, tissue inhibitor of metalloproteinase-3; TNF-α, tumor necrosis factor-α.

To visually integrate these interconnected signaling cascades and multi-target protective effects discussed above, we present a unified schematic summary summarizing the suggested synergistic network of exercise-mediated EV-derived ncRNAs in cardioprotection (**Figure 10**).

### 4.3 Research hotspots and future directions

#### 4.3.1 Research hotspots

##### 4.3.1.1 EVs as delivery vehicles for exerkines

The prominence of EVs in our analysis suggests their role as delivery vehicles for exerkines. Their lipid bilayer, surface proteins, and cargo composition may collectively contribute to their function as exercise-responsive delivery vehicles (Kong et al., 2026). Exercise may promote EV release and upregulates EV markers and skeletal muscle-related miRNAs, which may regulate skeletal muscle proliferation and differentiation (Estébanez et al., 2021). Different exercise intensities may induce EV release from endothelial cells, skeletal muscle, and adipose tissue. These EVs may exert anti-inflammatory and antioxidant effects through altered cargo composition (Shen et al., 2024). Together, EVs may combine structural stability, tissue origin diversity, and exercise-responsive sensitivity. These features may underpin the hypothesized exercise-EV-cardiovascular protection signaling axis.

##### 4.3.1.2 Exercise modality and differential EV cargo profiles

The range of exercise-related keywords points to the possibility that different modalities elicit distinct EV cargo profiles. A single session of blood flow restriction exercise has been suggested to alter surface marker profiles and miRNA cargo in circulating EVs. Five surface markers and 12 miRNAs showed significant post-exercise changes. Target prediction and functional enrichment suggest these miRNAs are predominantly associated with skeletal muscle protein metabolism (Just et al., 2020). In a resistance training model using skeletal muscle-specific Akt serine/threonine kinase 1 transgenic mice, growing muscle secreted EVs with elevated miR-1, miR-133, and miR-206. Among these, miR-206 may be taken up by endothelial cells and upregulate angiogenesis-related transcripts (Hayashi et al., 2024). Studies on aerobic exercise have largely focused on chronic adaptive effects of endurance training on circulating EV-derived miRNAs (Safdar et al., 2016). Aerobic and resistance training may differ in EV release, miRNA species, and targeted pathways, though direct comparisons are lacking.

##### 4.3.1.3 Inter-organ communication network of exercise-mediated EVs

Clusters involving interorgan communication and skeletal muscle adaptation highlight the skeletal muscle–cardiovascular axis as a potential research paradigm. Exercise has been suggested to promote the release of factors from the heart, liver, white/brown adipose tissue, and nervous system (Verboven and Vechetti, 2023). Studies suggest that skeletal muscle–derived EVs, myokines, and miRNAs mediate communication between skeletal muscle and the heart. Myokines such as follistatin-like protein 1, musclin, myonectin, and irisin may contribute to cardiovascular protection (Chen J. et al., 2025). It remains unclear whether exercise-mediated EVs participate in other organ crosstalk, such as adipose–heart, liver–heart, or gut–heart axes. Elucidating this interorgan network could offer insights into the cardioprotective mechanisms of exercise.

##### 4.3.1.4 Standardization of experimental techniques and methodologies

The presence of technical keywords in our burst analysis suggests increasing attention to mechanistic validation. However, the field currently faces challenges including a lack of uniformity in EV isolation and purification methods, inconsistency in miRNA detection standards, and substantial heterogeneity in data processing workflows (Yadav et al., 2024; Fazio et al., 2025). The complex composition of blood complicates EV isolation. Differences in isolation methods, characterization strategies, and exercise protocols across studies may limit comparability of results (Brahmer et al., 2020). The International Society for Extracellular Vesicles issued the Minimal Information for Studies of Extracellular Vesicles guidelines in 2014, 2018, and 2023 to promote standardization and reproducibility (Saint-Pol and Culot, 2025). However, these guidelines avoid prescribing specific methods, allowing laboratory variability (Pfaffl et al., 2025). This flexibility is scientifically justified, yet it may contribute to considerable methodological heterogeneity across studies, complicating direct comparisons and data integration. Achieving context-specific methodological consensus therefore remains a key priority for the field.

##### 4.3.1.5 Transition from animal models to human clinical validation

Burst analysis identified multiple translational research keywords, suggesting translational application as a potential trend. Currently, most evidence is derived from animal studies, while human studies remain limited. Existing human data suggest that concentrations of circulating EVs rise after exercise. These EVs, released from blood cells, skeletal muscle, and other exercise-related organs, carry bioactive cargo, suggesting they may mediate inter-tissue crosstalk during exercise (Llorente et al., 2024). Exercise-mediated EVs have also been shown to protect cardiomyocytes *in vitro*. EVs from human induced pluripotent stem cell-derived cardiomyocytes modulate electrophysiological properties under hypoxic stress, with preconditioned cells showing longer beating intervals and faster excitation–contraction coupling (Røsand et al., 2024). Although human studies and *in vitro* cardiomyocyte models have provided preliminary evidence supporting the cardioprotective effects of exercise-mediated EVs, the translation from animal models to human clinical validation remains at an early stage. Standardized clinical trial protocols and efficacy evaluation frameworks are needed to advance this field.

#### 4.3.2 Research trends

##### 4.3.2.1 Quantitative relationship between exercise dose and EV response

Despite growing interest in exercise modalities, the dose–response relationship between exercise intensity, frequency, and duration and circulating EV-derived ncRNAs expression profiles remains unclear. Emerging evidence suggests that acute and chronic exercise may modulate the release kinetics and cargo profiles of circulating EVs (Boulestreau et al., 2026). However, variations in exercise protocols complicate comparisons across studies (Maggio et al., 2023). Future large-scale cohort investigations are needed to construct an exercise dose-EV response model that could inform the optimization of individualized exercise prescriptions.

##### 4.3.2.2 Clinical translation of EV-derived ncRNAs as therapeutic targets

Translational keywords from our analysis suggest a shift toward clinical applications. Exercise-mediated EVs may serve as “exercise mimetics,” a cell-free therapeutic strategy for patients unable to exercise, by promoting EV secretion and inter-tissue communication. The cargo in exercise-mediated EVs may regulate metabolism and mitochondrial function, potentially improving cardiovascular outcomes (Wang et al., 2023). Combined application of mesenchymal stem cell-derived EVs and exercise may alleviate post-ischemic inflammation, oxidative stress, and apoptosis. This effect may involve inhibition of the extracellular signal-regulated kinase and AKT/mechanistic target of rapamycin pathways, offering insights into clinical translation (ShamsEldeen et al., 2025). Future studies should include well-designed, multi-center human exercise trials to assess whether exercise-mediated EV-derived ncRNAs could serve as biomarkers or therapeutic targets for CVDs.

##### 4.3.2.3 Application of artificial neural networks in exercise prescription optimization

The inclusion of “artificial intelligence” among our keywords suggests computational approaches may be entering this field. The EV response to exercise is influenced by multiple factors including age, sex, baseline fitness, and genetic background, and traditional statistical methods may be insufficient for dissecting their complex interactions (Garza et al., 2025; Kargl et al., 2024; McAdam et al., 2025). One study integrated transcriptomic data from acute myocardial infarction patients. It employed least absolute shrinkage and selection operator, random forest, and support vector machine recursive feature elimination for feature selection. This identified six EV-related diagnostic genes, including S100 calcium binding protein A9, MMP-9, and nucleotide-binding oligomerization domain-like receptor family pyrin domain-containing 3. A classification model for acute myocardial infarction was then built (Tang et al., 2026). Inspired by such analytical frameworks, artificial neural networks may offer a useful approach to disentangle multi-dimensional confounding variables and generate quantitative predictive frameworks, which could inform more precise, individualized exercise prescription design.

##### 4.3.2.4 Systematic integration of the multi-mechanism synergistic network

This cluster interconnection is in line with the proposed integrative nature of the multi-mechanism network. Exercise-regulated miRNAs have been reported to target multiple pathways, including PI3K/AKT, NF-κB, mitogen-activated protein kinase, and TGF-β/Smad3, potentially contributing to protection against apoptosis, oxidative stress, inflammation, and fibrosis (Lai et al., 2023). Future studies could further explore the crosstalk among these pathways and help identify shared upstream regulators, such as Nrf2 and NF-κB. Such efforts could contribute to establishing a conceptual foundation for developing multi-target intervention strategies.

##### 4.3.2.5 Interdisciplinary integration and technology-driven innovation

The citation flow observed in our dual-map analysis suggests a trend from basic research toward clinical application. Recent keyword trends suggest that the field may be moving from cardiovascular protection toward multi-system health regulation. For instance, the emergence of “cancer” suggests potential applications of exercise-derived EVs in tumor prevention and therapy (Ozerklig et al., 2025). The appearance of “body weight” may reflect a role for EVs in energy metabolism and weight regulation (Beckner et al., 2025). These trends suggest a shift from cardiovascular-specific protection to broader health regulation. Future research might bridge biomechanics, microfluidics, nanomedicine, exercise physiology and machine learning techniques. Cross-disciplinary integration of these fields could help decode the exercise-EV dose-response relationship and facilitate the translation of mechanistic findings into clinical intervention strategies.

##### 4.3.2.6 Translational challenges and barriers

Despite growing interest in translational applications, several notable challenges remain prior to their clinical translation. Delivering ncRNAs to target tissues can be challenging, as RNA molecules are generally unstable, nuclease-sensitive, and often poorly internalized due to their size and charge. Efficient loading, biodistribution control, and management of off-target effects add further complexity (Yang and Lv, 2026). Manufacturing scalability is another challenge. Current methods face challenges in maintaining batch-to-batch consistency and Good Manufacturing Practice compliance at scale. Automated bioreactor systems have shown promise, but standardized large-scale production protocols remain scarce (Li M. et al., 2025). Regulatory frameworks remain fragmented, and lacks globally harmonized standard (Limongi et al., 2026). Although clinical trials have grown, few EV-based products have received regulatory approval to date. This highlights the need for larger, well-controlled trials with standardized endpoints (Wei et al., 2026). Current clinical investigations of exercise-mediated EVs face challenges from small sample sizes, short-term intervention protocols, heterogeneous EV analytical pipelines, and a limited of evidence connecting exercise-mediated changes in EV-derived ncRNAs to cardiovascular hard endpoints (Llorente et al., 2024; Kargl et al., 2024). Overcoming these interconnected challenges will likely require coordinated advances in basic research, manufacturing, regulation, and trial design. These steps are important for advancing the therapeutic potential of exercise-mediated EV-derived ncRNAs.

### 4.4 Limitations

This study has several limitations. First, we used only three databases: WoSCC, Scopus, and PubMed. We excluded Embase because it overlaps substantially with Scopus and its pharmacological focus is less relevant. We also excluded Dimensions because its citation indexing and data export structure are less compatible with our software tools. Second, excluding conference abstracts, editorials, and non-English articles may underrepresent some frontier advances and regionally focused findings. Third, the high proportion of reviews in our sample may have introduced citation bias. This could amplify classical themes in the co-occurrence and clustering analyses and obscure emerging directions. Finally, despite manual merging of keywords, the interchangeable use of synonyms such as “exosome” and “extracellular vesicles,” as well as the heterogeneity in exercise intervention protocols and EV isolation methods across studies, may still introduce some interference in the interpretation of the co-occurrence network.

Beyond our bibliometric analysis, the broader field faces several challenges. These include heterogeneity in EV isolation and ncRNA characterization, limited human data compared with preclinical evidence, variability across experimental models, and obstacles in clinical translation. Addressing these issues will require standardized protocols, large-scale human trials, and improved regulatory frameworks.

## 5 Conclusion

Using 188 publications from WoSCC, PubMed, and Scopus (up to April 3, 2026), this bibliometric study outlines the knowledge structure and evolution of exercise-mediated EV-derived ncRNAs in cardiovascular protection. The main findings are as follows: (1) China and the United States are the leading contributors, together accounting for over 60% of publications, with China ranked first; (2) the research themes have undergone a three-phase evolution, from early dispersed exploration, to a mid-phase convergence on EVs and cardiovascular protection, and to a recent return to fundamental biology; (3) current hotspots include EVs as exerkine carriers, exercise-specific EV cargo, inter-organ communication, methodological standardization, and clinical translation; and (4) mechanistically, informed by bibliometric patterns and supported by published preclinical evidence, we propose that exercise may exert anti-apoptotic, antioxidant, anti-inflammatory, and anti-fibrotic effects by engaging a potential synergistic network involving EV-derived ncRNAs, though this framework requires experimental validation.

Future efforts should focus on: (1) establishing standardized protocols for the isolation and detection of EVs to support exercise-mediated EV research; (2) conducting large-scale human exercise trials to test the translational potential of preclinical findings; (3) integrating multi-omics and artificial intelligence to investigate the source heterogeneity and biomarker value of EV-derived ncRNAs; (4) promoting interdisciplinary integration to facilitate the translation of this field from mechanistic research to clinical application, offering new avenues for the early prevention and non-invasive intervention of CVDs; and (5) overcoming key translational barriers, including ncRNA delivery, scalable manufacturing, regulatory harmonization, and well-designed clinical trials with standardized endpoints, which will be important for clinical application.

## Data Availability

The original contributions presented in the study are included in the article/supplementary material. Further inquiries can be directed to the corresponding author.
